# A Rare Presentation of Scurvy in a Well-Nourished Patient

**DOI:** 10.7759/cureus.46379

**Published:** 2023-10-02

**Authors:** Connor J Robin, Kaleb J Robin, Mark A Maier, Elyse S Stevens

**Affiliations:** 1 School of Medicine, Louisiana State University Health Sciences Center, New Orleans, USA; 2 Department of Medicine, Louisiana State University Health Sciences Center, New Orleans, USA

**Keywords:** hemorrhagic disorders, purpura, malnutrition, ascorbic acid deficiency, clinical case report, vitamin c supplementation, malabsorption, vitamin deficiency, vitamin c, scurvy

## Abstract

Vitamin C deficiency, otherwise known as scurvy, is a rare diagnosis among populations with adequate nutritional resources. We present a 37-year-old female patient with bilateral lower extremity edema, episodic anasarca, petechiae, and easy bruising who was diagnosed with scurvy. Given the clinical presentation, a broad differential was investigated with no findings suggestive of hematologic or cardiovascular pathology. Initial laboratory studies were unremarkable. Progression of cutaneous symptoms and subsequent laboratory findings demonstrating low vitamin C levels supported a diagnosis of scurvy. Classical symptoms of scurvy include mucocutaneous petechiae, poor wound healing, ecchymosis, hyperkeratosis, corkscrew hair, gingival swelling, and bleeding gums. Following standard enteral supplementation of vitamin C, repeat vitamin C levels failed to adequately respond with the patient remaining to be symptomatic. Given a lack of insufficient nutritional intake or known systemic illness, gastrointestinal malabsorptive etiology was suspected. Though rare in the United States, scurvy should be considered in patients with manifestations of a bleeding disorder. A gastrointestinal workup may be indicated if other nutritional deficiencies are identified, or a source of inadequate intake cannot be established.

## Introduction

The prevalence of Vitamin C deficiency varies worldwide, with the United States reporting rates as low as 7% [1]. Humans lack the capacity to synthesize ascorbic acid, but this organic substance is essential to the human diet due to its role as a cofactor in many metabolic processes, the most important being collagen synthesis [2,3]. Procollagen alpha chains are abundant in the basal laminae of connective tissue, tunica media, and tunica adventitia of blood vessels, as well as osteoblasts that contribute to bone remodeling [2]. In individuals who eliminate foods high in ascorbic acid from their diet or suffer from malabsorptive gastrointestinal conditions, vitamin C deficiency can present with classical scurvy symptoms [2,4,5]. Without dietary ascorbic acid, the cofactor for lysyl hydroxylase and prolyl hydroxylase, collagen fibrils are not properly formed, leading to symptoms of deficiency. The onset of clinical symptoms usually occurs when total body storage levels of vitamin C are less than 300 mg for over one to three months [2,6]. Classical symptoms of scurvy include mucocutaneous petechiae, poor wound healing, ecchymosis, hyperkeratosis, corkscrew hair, gingival swelling, and bleeding gums [2,4,6]. Rheumatologic problems include muscle and soft tissue hemorrhaging and painful hemarthrosis.

Effective treatment of scurvy usually leads to symptomatic improvement with complete recovery within months of treatment onset [2]. Recent reports of patients with unresolved symptoms were found to have gastrointestinal comorbidities such as inflammatory bowel disease (IBD) and celiac disease (CD), suggesting a malabsorptive etiology of scurvy [5,7]. Although malabsorption may lead to a higher risk of scurvy, intravenous vitamin C supplementation and pharmacological treatment of the gastrointestinal disorder resolves symptoms in most patients [5].

## Case presentation

A 37-year-old female seeking the establishment of primary care presented to the integrated health clinic reporting a three-week history of non-blanching erythematous macules on the upper extremities and spontaneous ecchymoses on the abdomen and extremities. The patient reported that the lesions were not pruritic or painful, and the patient denied joint pain. The patient also described a one-year history of bilateral lower extremity edema with episodic anasarca and relative exercise intolerance. Past medical history was notable for severe opioid use disorder treated with a standard regimen of buprenorphine/naloxone, due to chronic musculoskeletal pain, leading to sustained remission for several years. The patient had no documented history of eating disorders or malnutrition. On the physical exam, bruising and generalized edema were present, and body mass index (BMI) was calculated at 29 kg/m^2^. Vital signs included blood pressure of 134/99, heart rate of 71 beats per minute, respiratory rate of 18 breaths per minute, and body temperature of 98 ^o^F (36.7 ^o^C).

Initial laboratory workup for a possible bleeding disorder revealed a normal platelet count (262,000 platelets/µL) with a low mean platelet volume at 6.5 fL. Other components of the complete blood count (CBC) were unremarkable. International Normalized Ratio (INR) and activated partial thromboplastin time (aPTT) were both normal at 1.0 and 27.1 s, respectively. The fibrinogen level was elevated at 627 mg/dL, and platelet function analysis was normal. The anasarca prompted further workup, including an echocardiogram which determined normal right and left ventricular function with a normal central venous pressure. Abdominal ultrasound exhibited no occlusion of the hepatic and portal venous systems. Lower extremity ultrasound was negative for deep vein thromboses (DVTs) bilaterally. Autoimmune and infectious etiologies were also explored, though the patient was determined to be hepatitis B virus (HBV) immune and had negative human immunodeficiency virus (HIV), antinuclear antibody (ANA), and hepatitis C virus (HCV) laboratory studies. Thyroid and adrenal studies were within normal limits. Protein electrophoresis was within the given reference ranges except for gamma globulin at 1.6 g/dL, just above the normal value of 1.5 g/dL. Protein electrophoresis was repeated one week later and found to be within normal limits. Furthermore, serum albumin was within normal limits at 4.2 g/mL.

At the 4.5-month follow-up, the patient reported symptom progression with the development of alopecia and brittle fingernails. Additionally, symptoms of arthralgias, bleeding gums, broken teeth, malaise, and depression prompted further investigation (Table 1). Laboratory workup revealed an elevation in c-reactive protein (CRP) to 5.8 mg/L with a normal erythrocyte sedimentation rate (ESR) of 17 mm/hr. Concern for autoimmune processes prompted antineutrophilic cytoplasmic antibody (ANCA) and anti-phospholipid analyses, which were both within normal limits. Quantification of vitamin levels revealed significantly decreased vitamin C at < 0.1 mg/dL (normal 0.4-2.0 mg/dL). 25-hydroxy vitamin D was also low at 13.2 ng/mL (normal 30-100 ng/mL), indicating moderate deficiency. Given the symptomatic vitamin C deficiency, a diagnosis of scurvy was established, and the patient was instructed to supplement her diet with 500-1,000 mg of vitamin C daily.

**Table 1:** Notable laboratory values near time of scurvy diagnosis. INR = international normalized ratio; aPTT = activated partial thromboplastin time.

Laboratory Study	Measured Value	Reference Range
Hematocrit	37.4%	35.0 - 46.0%
Platelet count	312 x 10^3^/µL	130 - 400 x 10^3^/µL
Mean platelet volume	7.8 fL	7.4 - 10.4 fL
Creatinine	0.97 mg/dL	0.5 - 1.1 mg/dL
25 hydroxy-Vitamin D	13.2 ng/mL	30 - 100 ng/mL
Vitamin C	< 0.1 mg/dL	0.4 - 2.0 mg/dL
Prothrombin time	11.4 seconds	10 - 13 seconds
INR	0.9	0.9 – 1.2
aPTT	27.1 seconds	24 - 37 seconds

The patient reported adherence to vitamin C supplementation at 1.5 months follow-up but continued to report alopecia as well as fatigue and hip pain. Vitamin C level had increased to 1.5 mg/dL, but vitamin D decreased further to 8.1 ng/mL. The patient was instructed to increase the vitamin C supplement to a minimum of 3 g daily as well as to begin vitamin D supplementation. Two months later, vitamin C level again decreased to 0.7 mg/dL near the lower limit of normal. Given the patient’s normal diet and adequate vitamin supplementation, other nutrient levels were considered for possible gastrointestinal etiology. Vitamins A, E, B6, B9, and B12 as well as zinc and copper were within normal limits. Gastroenterology consultation was initiated for further investigation of a possible malabsorptive nature of the nutrient deficiencies. Unfortunately, our patient was lost to follow-up due to unforeseen personal circumstances and resultant financial stressors.

## Discussion

Our patient developed symptoms and laboratory findings commonly seen in a scurvy diagnosis. Scurvy develops when plasma levels of vitamin C fall below 0.2 mg/dL, which typically occurs after one to three months of vitamin deprivation [2,4,6]. In developed countries where dietary sources of vitamin C are abundant, deficiencies are rarely found. However, studies have found that scurvy is more prevalent in populations with a high prevalence of psychiatric disorders, alcoholism, or ignorance of proper nutrition [2,6,8,9].

Our patient reported a balanced diet and adherence to vitamin C supplementation prior to and following the initial scurvy diagnosis. Recent literature has reported that patients who have gastrointestinal disorders such as IBD and primary sclerosing cholangitis are prone to nutritional deficiencies, one of those being vitamin C [5,8]. Without resolution of symptoms and persistently low vitamin C levels following several months of vitamin C supplementation, a gastrointestinal etiology is suspected following this patient’s presentation. In a recent retrospective cohort, it was reported that 21.6% of IBD patients had laboratory findings consistent with vitamin C deficiency [5]. Patients who have IBD frequently decrease nutritional intake or avoid certain food groups altogether. In addition, malabsorption and enteric loss of nutrients caused by the chronic release of inflammatory cytokines are also responsible for nutrient deficiencies found in IBD [8,10,11]. TNF-⍺ has been found to have profound effects on the major vitamin C intestinal transporter, SVCT-1, by inhibiting the protein, mRNA, and hnRNA expression levels of the transporter [11]. Our patient’s low serum levels of vitamin D could also be explained by an underlying gastrointestinal disease, as it has been reported that patients who have IBD have higher risk for concomitant nutritional deficiencies, including hypovitaminosis D [8,10]. IBD can cause malabsorption of bile salts, leading to impaired fat-soluble vitamin absorption [8,10].

Scurvy displays a wide spectrum of visible and radiologic findings that are helpful in diagnosis. Humans lack the capacity to synthesize ascorbic acid, but the organic substance is essential to the human diet due to its role as a cofactor to many metabolic processes, the most important being collagen synthesis [2,7,12]. Early manifestations of severe vitamin C deficiency include fatigue, decreased exercise tolerance, and depression [2,4,7,9]. The most distinguishing features of scurvy are cutaneous findings including follicular hyperkeratosis, perifollicular hemorrhages, ecchymoses, leg edema, and poor wound healing [2,4,9]. Oral findings involve interdental and marginal gingival erythema and swelling, as well as bleeding after minimal trauma [2]. Rarer presentations encompass conjunctival dryness, erythema, and irritation [2]. Chronic deficiency results in corkscrew hair and alopecia [2]. Laboratory levels of vitamin C can support the clinical diagnosis of scurvy [2].

When a patient presents findings of ecchymoses, petechiae, and mucosal bleeding, several potential etiologies should be assessed [13]. Abnormal bleeding could be caused by blood vessel disease, platelet disorders, or coagulopathies [13]. Blood vessel disease caused by trauma or chronic glucocorticoid use were largely excluded based on lack of history. Lack of sufficient dietary protein could have been a cause of poor coagulation factor production. However, our patient’s reported diet and adequate BMI did not support malnutrition due to lack of intake. Thus, our patient’s anasarca was not likely explained by poor oncotic pressure (albumin 4.2 g/dL) [14]. Systemic conditions, such as vasculitis or Cushing’s disease, were excluded due to negative ANCA and cortisol studies, respectively. Our patient also did not present with typical findings of genetic conditions such as Ehlers-Danlos syndrome, Marfan Syndrome, or osteogenesis imperfecta, which could cause similar presentations.

Platelet and coagulation factor deficiencies are also common causes of bleeding disorders. However, platelet count and platelet function analyses were all within normal limits. Liver disease, a cause of bleeding disorders and anasarca, was also not supported given ultrasound findings and negative viral studies. Furthermore, prothrombin time and partial thromboplastin time were normal, likely excluding genetic and nutritional causes of coagulopathies.

Other conditions were considered a cause of systemic edema as seen in our patient. Echocardiology findings and negative proteinuria were not supportive of cardiopulmonary etiology or nephrotic syndrome, respectively. Bilateral DVTs or chronic venous disease also do not typically have the presentation seen in our patient. With timely diagnosis and proper vitamin C supplementation, subjective improvement is reported within 24 hours, arthralgia and bleeding symptoms diminish within a week, and cutaneous and oral findings improve within two weeks to a month [2]. According to the Food and Nutrition Board (FNB), the Recommended Dietary Allowance (RDA) of vitamin C for all healthy individuals above the age of 19 years is 90 mg for males and 75 mg females [15]. Complete resolution of symptoms is usually seen within three months, while permanent findings can be limited to tooth loss [2]. In cases where gastrointestinal etiology is suspected and absorption of nutrients is compromised, oral supplement may prove ineffective, therefore intravenous administration is required to elevate and stabilize serum levels of ascorbic acid to its normal range [7,9].

## Conclusions

We present a rare case of scurvy nonresponsive to oral vitamin C intake within a well-nourished patient in an industrialized country. Though rare, practitioners should still consider scurvy in patients with typical findings of cutaneous and systemic signs of vitamin C deficiency. Gastrointestinal workup may be indicated to establish a cause for lack of sufficient vitamin levels if adequate dietary intake is suspected.
